# Simultaneous Analysis of Microsaccades and Pupil Size Variations in Age-Related Cognitive Impairment Using Eye-Tracking Technology

**DOI:** 10.3390/jemr19020029

**Published:** 2026-03-05

**Authors:** Seokjun Oh, Tahsin Nairuz, Sung-Jun Park, Jong-Ha Lee

**Affiliations:** 1 Department of Biomedical Engineering, Keimyung University, Daegu 42601, Republic of Korea; dk05056@naver.com (S.O.); tahsin.bmb@nstu.edu.bd (T.N.); 2 Department of Architectural Engineering, Keimyung University, Daegu 42601, Republic of Korea; sjpark@kmu.ac.kr

**Keywords:** Alzheimer’s disease, eye-tracking technology, microsaccades, pupil size variation

## Abstract

Age-related cognitive impairment represents a critical stage in the continuum of neurodegenerative disorders, including Alzheimer’s disease (AD), highlighting the need for objective and non-invasive physiological indicators of early neurological change. This study investigates the simultaneous analysis of microsaccadic eye movements and pupil size variations as ocular biomarkers associated with age-related cognitive impairment using eye-tracking technology. A total of 70 participants were recruited and categorized into three age groups: individuals in their 20s, 60s, and 70s. Participants in their 70s were further categorized based on MMSE-K scores into cognitively normal (≥24) and impaired (≤23) subgroups. Quantitative analyses showed a significant age-related increase in microsaccade frequency along both axes, with significantly higher microsaccade frequencies (*p* < 0.01) among individuals with lower cognitive scores within the same age group. Pupil size variation, including constriction and dilation rates, declined with age, while response speed remained relatively unchanged across all age groups. These findings highlight a clear association between age related-cognitive decline and involuntary ocular responses. The proposed dual-biomarker method offers a non-invasive and quantitative framework that may complement traditional cognitive screening tools. Future studies involving larger cohorts and clinically diagnosed AD populations are required to determine the diagnostic utility of these ocular biomarkers.

## 1. Introduction

Alzheimer’s disease (AD) represents the most prevalent form of major neurodegenerative dementia, accounting for around 60–80% of all dementia cases [1]. Clinically, AD is characterized by a progressive and irreversible decline in cognitive function, underpinned by neuronal loss and pathological alterations in brain tissue [2]. While episodic memory impairment is a hallmark feature, the disease is also frequently accompanied by several cognitive impairments, including executive functioning, language, praxis, abstract reasoning, problem-solving, and visuospatial perception impairments, ultimately impeding the ability to perform basic and instrumental activities of daily living [3,4,5,6,7].

The onset of AD symptoms is usually subtle, beginning with mild memory disturbances that gradually turn into more pervasive cognitive deficits. Moreover, significant functional dependence and physical decline occur in later stages. Although the rate of disease progression varies among individuals, the average life expectancy is estimated to range between 3 and 9 years following a formal diagnosis [8,9].

In AD, early diagnosis and intervention are crucial for slowing disease progression, enhancing quality of life, and potentially extending survival [10]. However, early detection remains a significant challenge, as initial symptoms appears slowly and are frequently misinterpreted as normal aging in up to 50–70% of cases, leading to delayed diagnosis [4,5,10]. To address this diagnostic gap, efficient, reliable, and accessible early detection systems are necessary for identifying individuals at risk before substantial cognitive decline occurs. Early identification is also essential for maximizing the effectiveness of future disease-modifying therapies.

Currently, neuropsychological assessments such as the Mini-Mental State Examination (MMSE) and the Montreal Cognitive Assessment (MoCA) are widely employed as standard tools for evaluating cognitive function in individuals with Alzheimer’s disease [11]. While these methods serve as primary diagnostic frameworks in clinical practice, their limitations are significant. Specifically, they require professionally trained medical personnel and are time-consuming. Studies have shown that their proper administration and interpretation require training in clinical neuropsychology, and administration times can range from 10 to 15 min or more, depending on patient condition and cognitive level [11,12]. Moreover, both tools may require additional time for explanation, scoring, and follow-up in cases of borderline or ambiguous results [13]. In addition, their results can be influenced by external factors such as patient anxiety or emotional state, potentially compromising the objectivity of the results. Furthermore, these tests often exhibit educational bias, which can affect the reliability of results for individuals whose educational background differs from that of the general population [14,15].

In contrast to traditional neuropsychological assessments, eye-tracking technology provides a non-invasive, objective, and efficient alternative [16]. It eliminates the need for active behavioral responses, such as speech or motor input, thus reducing cognitive and physical demands on patients [17]. Modern eye-tracking systems, with their high temporal resolution (up to 1000 Hz), can rapidly gather extensive time-series data, which enables in-depth analysis of visual and cognitive processing [18]. Recently, the growing accessibility of eye-tracking technology, along with the simplicity of its instrumental requirements, has facilitated its integration into clinical research, especially studies involving cognitively impaired populations [19,20,21,22,23].

Although eye-tracking has been widely employed to investigate higher-order cognitive processes in cognitive psychology [24,25], its application in dementia research remains relatively limited. However, studies by Crutcher et al. [26] and Richmond et al. [27] are notable exceptions as they utilized visual paired-comparison tasks to show that eye movement parameters—such as fixation count and duration—can reliably distinguish individuals with mild cognitive impairment (MCI) from cognitively healthy controls, reflecting underlying short-term memory deficits. Similarly, Fernández et al. [28] examined semantic, working, and retrieval memory impairments in individuals with young-onset Alzheimer’s disease by analyzing eye movement differences during the reading of high- versus low-predictability sentences. These findings demonstrate the untapped potential of eye-tracking metrics as early indicators of cognitive dysfunction in Alzheimer’s and related disorders.

Recent advancements in non-invasive diagnostic methodologies have increased in identifying ocular biomarkers associated with age-related cognitive impairment, using visual metrics, such as pupil size measurement, brain protein observation via the pupil, retinal vascular density analysis, and eye movement analysis. In particular, the quantitative evaluation of microsaccade patterns has gained increasing attention from both research institutions and companies as a promising, low-cost, and minimally burdensome alternative to conventional cognitive assessments. Microsaccades, the largest and fastest eye movements occurring during attempted fixation, are subtle, involuntary motions that reflect underlying neurocognitive processes [29] and have been shown to exhibit altered patterns in a range of neurological disorders, including Alzheimer’s disease, Parkinson’s disease, and ADHD [30]. While key microsaccadic parameters such as amplitude, velocity, duration, and inter-microsaccadic intervals remain preserved in AD, patients with MCI and early Alzheimer’s consistently demonstrate a higher frequency of oblique microsaccades compared to healthy individuals [31].

In parallel, pupil size variation reflects a distinct yet complementary neurophysiological pathway. Pupil constriction and dilation are regulated by the autonomic nervous system, with the parasympathetic (cholinergic) system stimulating constriction and the sympathetic (adrenergic) system inducing dilation. Age-related degeneration of cholinergic and noradrenergic systems, which is well documented in cognitive aging and Alzheimer’s disease-related pathology, has been associated with reduced pupillary responsiveness and altered pupil dynamics [32,33,34]. Importantly, pupil size variation captures global autonomic and neuromodulatory function and has been shown to decline progressively with advancing age, even in cognitively healthy populations, making it a relevant physiological indicator of age-related neural change.

From a neurophysiological perspective, microsaccades and pupil size variation capture complementary dimensions of brain function. Microsaccades primarily index cognitive–oculomotor control and attentional regulation, whereas pupil dynamics reflect autonomic and neuromodulatory integrity. Integrating these two ocular biomarkers within a single eye-tracking framework therefore enables simultaneous assessment of both cognitive and autonomic components of neural function, offering a more comprehensive characterization of age-related cognitive impairment than either measure alone. Such a dual-biomarker approach is particularly valuable in research on cognitive aging and Alzheimer’s disease-related processes, where early neurophysiological changes may be subtle, multifactorial, and distributed across neural systems.

Although prior studies have independently explored either microsaccades or pupil size dynamics as potential indicators of cognitive decline, to the best of our knowledge, no studies have yet combined these two metrics in a unified experimental framework. To address this gap, the present study quantitatively measures and analyzes microsaccadic pattern and pupil size variation across different age groups, with the aim of characterizing age-related changes in ocular dynamics and their association with cognitive status Through this dual-biomarker analysis, this study addresses key limitations of traditional cognitive assessments, such as the MMSE and MoCA, by offering a rapid, objective, and quantitative approach that reduces dependence on verbal or task-based responses, and supports the assessment of age-related cognitive impairment, with potential relevance for future Alzheimer’s disease research.

## 2. Materials and Methods

### 2.1. Study Subjects

A growing body of neurocognitive research has consistently demonstrated that the risk of cognitive decline becomes increasingly pronounced with advancing age, particularly as individuals progress from their 50s into their 60s. Notably, individuals aged 75 years and older exhibit a 1.75-fold increased risk of cognitive impairment relative to those aged between 65 and 74 years [35]. Building upon this evidence, the present study stratified participants into three distinct age cohorts: individuals in their 20s, those in their 60s, and those in their 70s—the younger group serving as a reference for age-related comparisons and the latter two age groups representing increased susceptibility to age-related cognitive decline.

The study subjects comprised a total of 70 participants: 30 individuals aged in their 20s, 10 in their 60s, and 30 in their 70s. Age is reported as mean ± standard deviation (years), with mean ages of 24 ± 2 years for the 20s group, 63 ± 5 years for the 60s group, and 74 ± 4 years for the 70s group. The sex distribution was as follows: in the 20s group, 15 males and 15 females; in the 60s group, 4 males and 6 females; and in the 70s group, 12 males and 18 females. Participants were recruited from the community through general advertisements and were not selected based on self-reported cognitive concerns or family history of cognitive impairment; objective cognitive status was assessed only after enrollment using the Korean version of the Mini-Mental State Examination (MMSE-K). Within the 70s cohort, participants were further categorized based on MMSE-K scores: individuals scoring ≥24 were classified as “cognitively normal” (*n* = 14), and those scoring ≤23 fell into the “suspected dementia” classification (*n* = 16). Within these subgroups, the cognitively normal group consisted of 6 males and 8 females, whereas the low-scoring subgroup included 6 males and 10 females. This classification is aligned with and validated by previous studies conducted in community-dwelling elderly populations in Korea [36]. Specifically, a study by Lee et al. proposed that MMSE-K scores ≥24 indicate normal cognition, 18–23 indicate mild cognitive impairment, and ≤17 indicate severe cognitive impairment [37]. However, this classification reflects cognitive screening outcomes only and does not constitute a formal clinical diagnosis.

All participants were screened by self-report for major ophthalmic, neurological, and psychiatric conditions that could affect eye-tracking or pupillary measurements, as well as for the use of medications known to significantly influence pupillary function; no such conditions or medications were reported. Participants requiring optical correction were permitted to wear their usual correction during testing. The study was approved by the Keimyung University Institutional Review Board (IRB No. 40525-202211-HR-061-03), and written informed consent was obtained from all participants prior to participation.

### 2.2. System Setup and Eye Tracking

**Figure 1:**
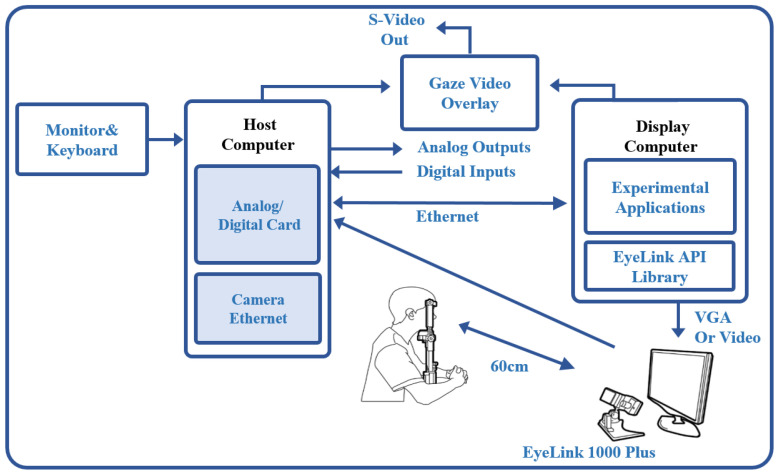
Experimental setup using Eyelink 1000 plus for eye-tracking and data collection.

In this study, an experimental setup was designed to simultaneously measure microsaccadic eye movements and pupil size variations using eye-tracking technology. The system consisted of two interconnected computers and an eye-tracking camera. The first computer, designated as the Host Computer, was exclusively tasked with data collection. The second computer, referred to as the Display Computer, was responsible for creating and executing experimental files via Experiment Builder software, serving as the medium through which stimuli were presented to the participants. The Display Computer was primarily used to present stimuli to participants. The two computers were connected via an Ethernet link, allowing the Host Computer to share critical information, such as pupil-related events, gaze position, and camera images, with the Display Computer throughout the experiment. The schematic representation of the experimental setup, as depicted in Figure 1, outlines the system’s configuration and operational design.

Each participant was positioned at a fixed distance of 60 cm from the camera (Eyelink 1000 Plus, SR Research Ltd., Ottawa, ON, Canada) and was carefully stabilized to prevent any pupil displacement caused by body movements, thus ensuring the integrity of the eye-tracking data. All experimental sessions were conducted in a completely dark, noise-free room, with all lights turned off to avoid any external visual interference that could alter pupil size or eye movement behavior. A standard 9-point calibration was performed on the Display Computer screen at the beginning of each session. Calibration was repeated as necessary until validation confirmed that the alignment error across all nine points remained below 1.0°, ensuring precise eye-tracking accuracy.

Following successful calibration and validation, participants performed a passive fixation task, in which they were instructed to maintain a steady gaze on a stationary central fixation point (5 mm in diameter) displayed on the screen. Visual stimuli were presented on a 24-inch LCD monitor (Dell P2419H, Dell Inc., Round Rock, TX, USA) with a native resolution of 1920 × 1080 pixels and a refresh rate of 60 Hz, operated in standard LCD mode with factory-calibrated color settings. The bright screen consisted of a uniform white stimulus (RGB: 255, 255, 255) with a measured luminance of 120 cd/m^2^, whereas the dark screen consisted of a uniform black stimulus (RGB: 0, 0, 0) with a measured luminance of 0.5 cd/m^2^. Luminance was measured at the center of the display using a calibrated photometer.

No cognitively demanding tasks or additional stimuli were introduced during the experiment to isolate involuntary ocular responses, such as microsaccades and pupil size changes, without cognitive interference. To assess pupil constriction and dilation, participants were presented with a visual stimulus repeating five times, each consisting of an 8 s bright screen followed by an 8 s dark screen, while continuously fixating on the central point. This yielded a total of 80 s of pupillometry data per participant. Microsaccades and pupil size variations were recorded simultaneously during this single-fixation-based experimental paradigm, and microsaccadic events were extracted offline from the same eye-tracking recordings obtained during the pupillometry protocol. Microsaccadic movements were analyzed during steady-fixation periods while minimizing voluntary ocular movements, and to reduce potential influences of luminance transitions on oculomotor behavior, data segments immediately following bright–dark or dark–bright screen transitions were excluded from microsaccade analysis. The eye-tracking camera was configured to record binocular eye movements at a sampling rate of 500 Hz, with three separate trials conducted for each participant. Although the system supports sampling rates up to 1000 Hz, 500 Hz was selected because it provides sufficient temporal resolution for reliable detection of microsaccades and pupillary dynamics while ensuring stable data acquisition and reducing susceptibility to signal loss, particularly in elderly participants. These trials made it possible to accurately capture both pupil size fluctuations and microsaccadic movements. Only movements occurring within a 2° radius of fixation were classified as microsaccades, while deviations exceeding this threshold were regarded as extraneous noise and were subsequently excluded from the data analysis.

### 2.3. Pupil Size Unit Conversion

When pupil size data is collected through the Eyelink 1000 Plus, the measurements are initially recorded in arbitrary units, requiring a conversion into millimeters for analysis. Due to errors arising from variations in the distance and angle between the camera and the participant’s pupils, the actual sizes of the left and right pupils may appear different. Therefore, it is necessary to compute a distinct scaling factor for each eye. To determine the scaling factor, a specific code is added to the Eyelink Host Computer to switch it to artificial eye measurement mode. Once this mode is activated, an artificial eye with a known diameter is positioned for measurement. In this experiment, a 6 mm artificial eye was utilized for this purpose. Following the measurement process, the pupil size recorded in arbitrary units is then converted into millimeters to calculate the scaling factor using the following formula:

$$\mathrm{Scaling}\mathrm{Factor}=6\;\mathrm{mm}/\sqrt{\left(pupil\right)}$$

As a result of this calculation, the scaling factor for the left eye was determined to be 0.1188, while the right eye’s scaling factor was 0.1126. After obtaining these values, the code that switched the system to artificial eye measurement mode was deactivated to exit this mode. Subsequently, the actual pupil size of the participants was measured, with the following formula applied to convert the measured pupil data into millimeters:

$$\mathrm{Pupil}\mathrm{in}\mathrm{mm}=\mathrm{Scaling}\mathrm{Factor}\times \sqrt{\left(pupil\right)}$$

To quantify the dynamics of pupil size changes, we calculated the constriction rate and dilation rate based on the magnitude of pupil size variations in response to alternating light stimuli. The constriction rate represents the percentage reduction from baseline pupil size to the minimum size after exposure to a bright screen. The dilation rate reflects the percentage increase from the minimum size to the maximum size following a dark screen. These were computed using the following formulas:

ConstrictionRate (%)=[(baselinesize−minimumsize)/baselinesize]×100

DilationRate (%)=[(maximumsize−minimumsize)/minimumsize]×100

### 2.4. Statistical Analysis

All statistical analyses were performed using IBM SPSS Statistics version 20 (IBM Corp., Armonk, NY, USA). To examine group differences across age and cognitive status cohorts, one-way analysis of variance (ANOVA) was conducted specifically for microsaccade frequency metrics. When significant effects were detected, post hoc comparisons were performed using the Tukey method to control for Type I error due to multiple comparisons. Prior to conducting ANOVA, assumptions such as normality and homogeneity of variances were assessed to ensure the validity of the tests. Data are expressed as mean ± standard deviation (SD), and statistical significance was set at ** p < 0.01.

## 3. Results

### 3.1. Analysis of Microsaccades

**Figure 2:**
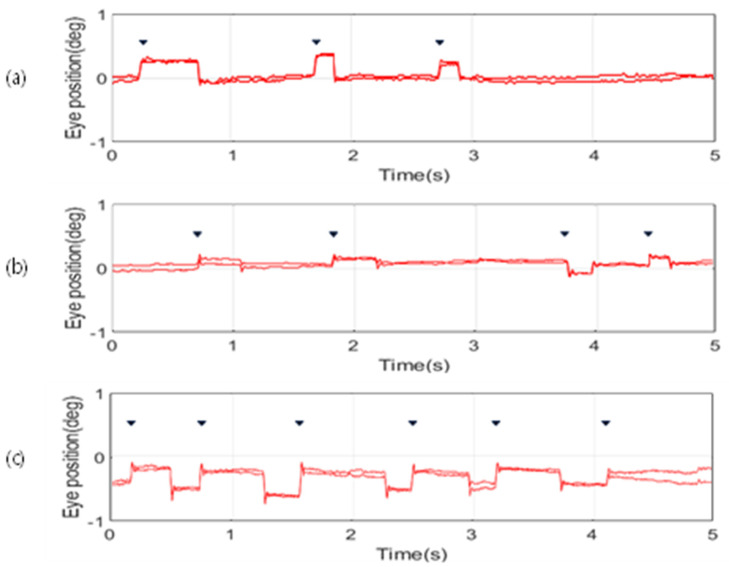
X-axis pupil movement in both eyes for participants in (**a**) their 20s, (**b**) their 60s, and (**c**) their 70s, with microsaccadic events marked by downward triangles.

In this study, the movement data of the pupil along both the x-axis and y-axis for participants in their 20s, 60s, and 70s were visualized in graph form to analyze the occurrence of microsaccades. Figure 2 depicts a graphical representation generated using the Eyelink Data Viewer, depicting the x-axis movements and microsaccadic occurrences across both eyes for each age group. Younger participants (20s) exhibit stable and infrequent microsaccades, while those in their 60s show an increase in microsaccadic occurrences, suggesting early age-related changes in oculomotor control. Participants in their 70s display even more frequent and variable microsaccades, indicating increased complexity and variability in fixation eye movements with advancing age.

**Figure 3:**
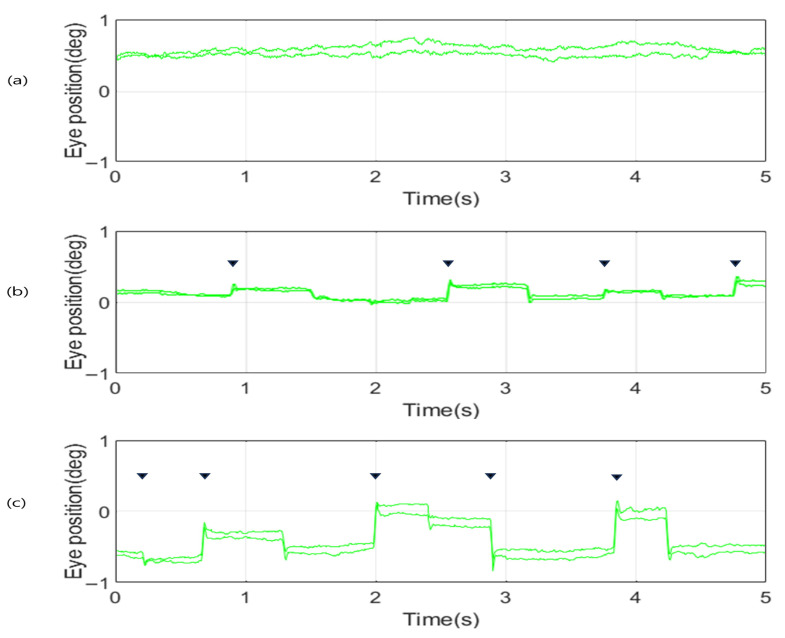
Y-axis pupil movement in both eyes for participants in (**a**) their 20s, (**b**) their 60s, and (**c**) their 70s, with microsaccadic events marked by downward triangles.

Further analysis, as shown in Figure 3, illustrates the y-axis pupil movements and microsaccade occurrences in both eyes, visualized using the Eyelink Data Viewer. In the 20s cohort, microsaccades along the y-axis were either minimal or occurred at a markedly lower frequency, suggesting that pupil movements remained relatively stable and less dynamic. In contrast, participants in their 60s and 70s exhibited more frequent microsaccades along the y-axis, with an expanded range of movement compared to their younger counterparts. These observations suggest that with increasing age, the complexity of pupil movements escalates, with microsaccades occurring along both the x-axis and y-axis.

The following step in the analytic pipeline involved the adaptation and refinement of open-source RStudio (version 2023.12.1) code from the Eyelink SR-Research platform in conjunction with the Microsaccades Toolbox, enabling the visual transformation and systematic analysis of binocular microsaccade data across participant age cohorts. This methodology facilitated the delineation of age-specific microsaccade patterns and provided a nuanced visualization of pupil movement dynamics, allowing for the identification of characteristic age-related changes in involuntary eye behavior.

**Figure 4:**
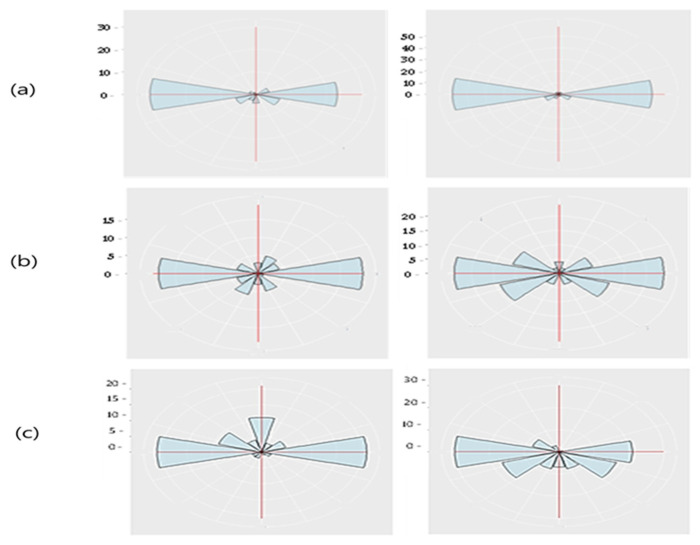
Polar plots of microsaccade directions for participants in (**a**) their 20s, (**b**) their 60s, and (**c**) their 70s.

As depicted in Figure 4a, the microsaccadic patterns observed among participants in their 20s were predominantly constrained to horizontal trajectories along the x-axis. This spatial confinement suggests a relatively stable and stereotyped microsaccadic profile, indicative of efficient ocular motor control and low variability in involuntary eye movement during fixation. Such findings underscore the simplicity and predictability of microsaccades in early adulthood. In contrast, the microsaccade distributions in participants aged in their 60s and 70s, visualized in Figure 4b,c, exhibited horizontal movement patterns similar to those of the 20s group. However, unlike the younger participants, whose microsaccades were strictly horizontal, older participants also displayed vertical movements. Moreover, these older age groups exhibited a broader dispersion of microsaccade, reflecting increased variability and less spatial constraint in ocular micro-movements. These patterns suggest a possible age-related decline in the precision of oculomotor regulation.

**Figure 5:**
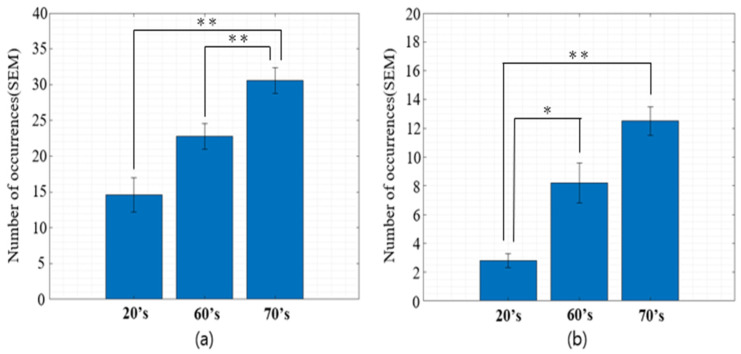
Number of microsaccade occurrences across different age groups. (**a**) Horizontal (x-axis) microsaccades; (**b**) Vertical (y-axis) microsaccades. Data represent the average number of occurrences per age group (20s, 60s, and 70s). Significant differences between groups are indicated: * *p <* 0.05, ** *p* < 0.01.

To extend the investigation of age-related alterations in fixational eye movements, a comparative analysis was conducted to quantify the frequency of microsaccade occurrences across different age groups. As illustrated in Figure 5, the frequency of microsaccades along the x-axis exhibited a significant increase from the 20s through the 60s to the 70s cohort. In participants in their 20s, horizontal microsaccades occurred with relatively low frequency; however, this pattern shifted significantly with age: the 60s group showed a significant elevation in microsaccade frequency, which further significantly increased in the 70s group, where the highest occurrence was recorded compared to the 20s group. Subsequently, the frequency of microsaccades was assessed along the y-axis. In the 20s group, microsaccades were rarely observed. However, this pattern evolved with age, as shown by a significant increase in microsaccade frequency in the 60s group and a substantially pronounced elevation in the 70s cohort compared to the 20s group.

**Figure 6:**
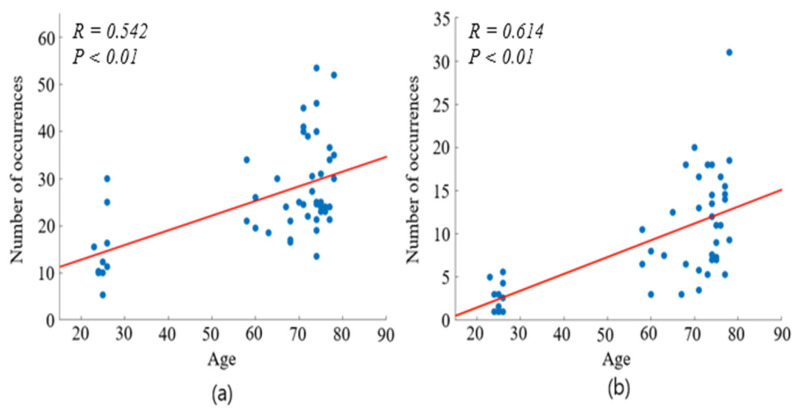
Correlation between age and the number of microsaccade occurrences. (**a**) Horizontal (x-axis) microsaccades; (**b**) Vertical (y-axis) microsaccades. Pearson correlation analysis was performed. The red line represents the linear regression fit. Correlation coefficients and *p*-values are indicated: (**a**) R = 0.542, *p* < 0.01; (**b**) R = 0.614, *p* < 0.01.

To visualize these trends collectively, Figure 6 presents a comparative graph plotting the frequency of microsaccades along both axes as a function of age. The plotted trajectories exhibit a clear linear progression, indicating that microsaccade frequency increases with age. Pearson correlation analysis revealed a positive correlation between chronological aging and microsaccade occurrence in both horizontal and vertical dimensions (R = 0.542, *p* < 0.01, R = 0.614, *p* < 0.01).

Building upon the observed age-related shifts in microsaccadic frequency and directional dispersion, the present study further investigated the role of cognitive status as a modulating factor in these involuntary oculomotor behaviors. Specifically, to assess the influence of cognitive decline on microsaccades, a targeted comparison was conducted between participants in their 20s and those in their 70s. Within the 70s cohort (N = 30), cognitive performance was evaluated using a standardized cognitive assessment tool. A total of 14 individuals who scored 24 or higher were classified as cognitively normal, while the remaining 16 participants, who scored 23 or lower, were categorized within the “suspected dementia” range—indicative of potential neurodegenerative impairment.

**Figure 7:**
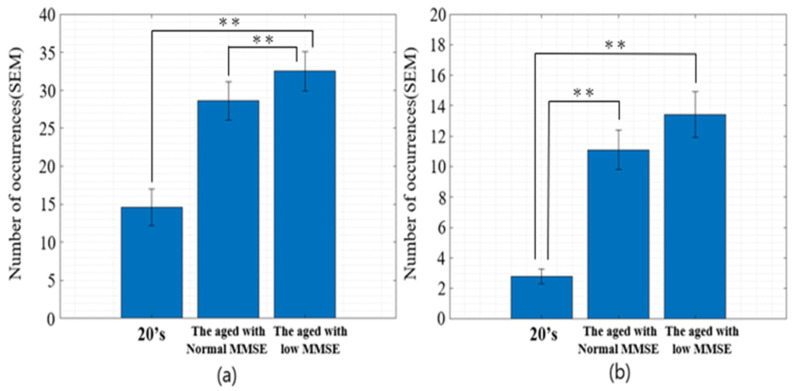
Average number of microsaccade occurrences along the (**a**) horizontal (x-axis) and (**b**) vertical (y-axis) directions for three groups: participants in their 20s, elderly participants with normal MMSE scores (≥24), and elderly participants with low MMSE scores (≤23). Significant differences between groups are indicated: ** *p* < 0.01.

As depicted in Figure 7, participants in their 20s demonstrated the lowest frequency of microsaccades along both the x-axis and y-axis. However, within the 70s group, a distinct bifurcation emerged: individuals with lower cognitive scores exhibited a significantly higher frequency of microsaccades compared to their cognitively normal counterparts, despite being of the same age range. This divergence highlights that cognitive function, independent of age, plays a critical role in modulating the frequency and variability of microsaccadic events.

### 3.2. Analysis of Changes in Pupil Size

**Figure 8:**
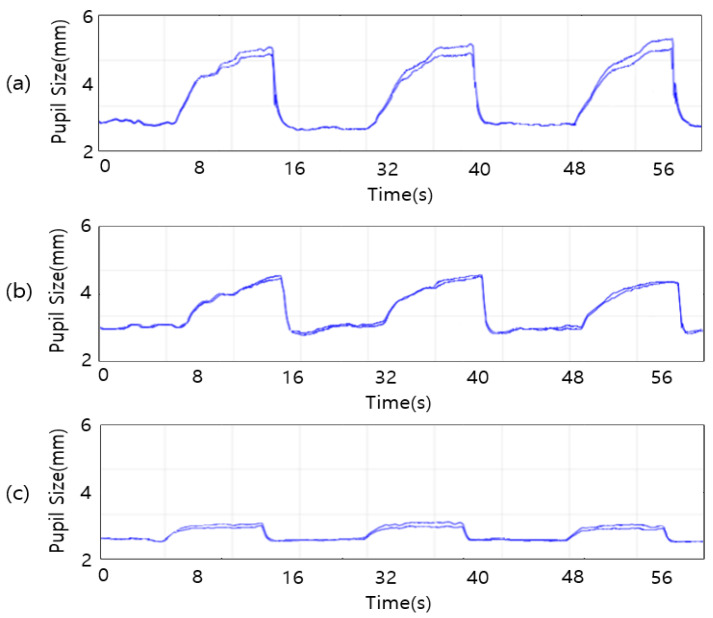
Binocular Pupil Dilation and Constriction Amplitude: (**a**) 20s, (**b**) 60s, (**c**) 70s Age Groups.

In this study, variations in pupil size were assessed among participants in their 20s, 60s, and 70s to examine the effects of aging on pupil dynamics, with analyses focused on age-group comparisons rather than cognitive-status subgroup comparisons. Using the Eyelink Data Viewer, key metrics—including constriction rate, dilation rate, and overall response speed—were compared across the age groups. As illustrated in Figure 8, the results revealed a clear age-related decline in the amplitude of pupil size variations. Notably, the constriction and dilation rates were highest in the 20s group. In contrast, participants in their 70s exhibited a significantly lower pupil response amplitude, suggesting that pupil responses diminish with aging. Despite this decline in response magnitude, no substantial differences in pupil constriction and dilation speeds were observed across all age groups.

Table 1 further represents age-related attenuation in pupillary response by detailing the constriction and dilation rates across different age groups. Participants in their 20s displayed the highest levels of responsiveness, with an average constriction rate of −51.5579 and a dilation rate of 114.9462, underscoring a robust capacity for pupil size variation in response to visual stimuli. In contrast, individuals in their 60s exhibited a noticeable decline, with constriction and dilation rates reduced to −43.685 and 80.3637, respectively. The most pronounced reduction was observed in the 70s group, where the average constriction rate fell to −38.3559 and dilation rate to 63.4061. This progressive decline in both metrics across age groups highlights a clear decline in pupil response with advancing age.

Moreover, Table 2 shows the timing of pupil responses—specifically, the latency of constriction and dilation. The average constriction time for participants in their 20s, 60s, and 70s consistently measured around 1.22 s, while the dilation time held steady at approximately 7.6 s across all groups. Even within the 70s group, the measured constriction and dilation times—1.13 s and 7.49 s—remained largely unchanged with aging. These results indicate that pupil response speed is not significantly affected by age and that the pupil reacts at a consistent rate over time. This suggests that the neural timing mechanisms responsible for initiating and completing pupil responses are preserved despite an age-related decline in autonomic response.

## 4. Discussion

This study examined microsaccades and pupil size variations as non-invasive physiological markers of age-related cognitive impairment, investigating how aging and cognitive function changes influence these ocular responses. By analyzing participants across three age groups (20s, 60s, and 70s) and incorporating cognitive assessments, the study aimed to characterize the combined effects of aging and cognitive decline on fixational eye movements and pupillary dynamics. Through the integration of eye-tracking data with cognitive performance measures, this research focused on elucidating age-related changes in neuromotor and autonomic visual systems and exploring their potential relevance to early neurocognitive changes associated with vulnerability to neurodegenerative disorders, including Alzheimer’s disease.

The findings revealed a significant age-related increase in microsaccade frequency, with particularly pronounced changes observed in the vertical (y-axis) direction. Participants in their 20s exhibited low microsaccade frequency with predominantly horizontal orientation, reflecting stable and efficient oculomotor control. In contrast, older participants, especially those in their 70s, showed not only significantly increased microsaccade frequency but also greater directional dispersion—including a significant rise in vertical microsaccades. This effect was further intensified in elderly participants with lower cognitive scores, suggesting that age-related decline in attentional regulation and motor control systems may contribute to increased microsaccadic instability. These observations are consistent with previous studies reporting altered microsaccade patterns in individuals with cognitive impairment and age-related neurodegenerative conditions [30], supporting the potential role of microsaccades as physiological indicators of age-related cognitive decline.

In parallel, pupil size variation analysis revealed a gradual decline with advancing age. Specifically, both constriction and dilation rates progressively decreased from the 20s to the 70s group, suggesting a reduction in the dynamic range of the pupillary response. These age-related reductions are consistent with prior evidence demonstrating a progressive decline in pupil size dynamics across the healthy lifespan, reflecting age-related changes in autonomic and neuromodulatory function [38]. These findings are further supported by previous studies examining pupil constriction and dilation responses to light stimuli in both healthy individuals and individuals with Alzheimer’s disease, confirming that aging significantly affects pupillary responsiveness [39]. Notably, the diminished amplitude of pupil size variation suggests a potential link to age-related decline in neural function and may represent an indicator of reduced visual and autonomic response capability in older adults. Given that aging is a primary risk factor for cognitive decline and neurodegenerative disorders, the present findings support the interpretation that pupil size variation reflects aging-related neurophysiological changes relevant to cognitive impairment.

However, unlike the changes observed in response amplitude, the speed of pupil constriction and dilation remained relatively unchanged across all age groups. Even among the oldest participants, pupil response time showed no significant difference compared to younger cohorts. This preservation of reflexive timing, despite diminished response magnitude, suggests that while the strength of the autonomic response declines with age—potentially due to reduced parasympathetic and sympathetic nervous system function—the underlying neural timing mechanisms remain functionally intact. In addition to age-related changes observed in our study, prior research on the pupillary light reflex (PLR) provides important context for interpreting pupillometry findings in both normal aging and Alzheimer’s disease research. Quantitative PLR studies in aging populations have documented systematic alterations in constriction and dilation dynamics, including reduced constriction amplitude and slowed response timing, which are interpreted as reflecting autonomic and neuromodulatory changes with advancing age [40]. In Alzheimer’s disease cohorts, recent work using quantitative light reflex pupillometry (qLRP) has demonstrated diagnostic potential, with altered constriction and recovery patterns differentiating AD patients from healthy controls and indicating their value as non-invasive digital biomarkers of neurodegeneration [41]. Although many of these studies employ dedicated pupillometry devices, the core parameters analyzed—such as amplitude, latency, and dynamic response characteristics—are conceptually and quantitatively comparable to those obtainable via high-resolution eye-tracking systems. This body of evidence reinforces the relevance of pupillometry as a sensitive indicator of autonomic and neurophysiological changes across cognitive aging.

The convergence of these two physiological markers—microsaccades and pupil size variation—offers valuable insight into how neurodegenerative processes influence involuntary eye behavior. Microsaccadic eye movements are controlled by a network that includes the superior colliculus, frontal eye fields, parietal cortex, and basal ganglia—regions responsible for visual fixation, attentional shifts, and oculomotor inhibition [42,43,44]. Neurodegeneration in these areas, particularly the frontoparietal cortex, can disrupt oculomotor function and inhibitory control, potentially leading to the increased frequency and spatial irregularity of microsaccades seen in early AD [31]. Consequently, pupil size fluctuations reflect the dynamic interplay of the autonomic nervous system, particularly the parasympathetic (cholinergic) and sympathetic (adrenergic) pathways [45]. Pupil dilation is regulated by the locus coeruleus–norepinephrine (LC–NE) system, one of the first sites to show tau pathology in preclinical AD. Degeneration in this system reduces pupil dilation capacity and impairs arousal-linked autonomic responses [46]. Pupil constriction is mediated by the Edinger–Westphal nucleus, which receives cholinergic input from the basal forebrain—another region vulnerable to early AD pathology [47]. Cholinergic decline contributes to the diminished amplitude of pupil constriction, a feature observed in AD patients [34]. Therefore, disruptions in both attentional and autonomic systems may manifest as abnormal microsaccadic and pupillary responses, supporting the biological plausibility of these physiological markers as indicators of early cognitive dysfunction.

Importantly, the results of this study suggest that both microsaccade and pupil dynamics could serve as sensitive, non-invasive biomarkers for early cognitive decline. These metrics enable the detection of subtle yet measurable differences between cognitively normal and impaired individuals within the same age group, offering significant promise for preclinical screening and longitudinal monitoring. Compared to traditional neuropsychological assessments, eye-tracking and pupillometry offer advantages in terms of objectivity, cost-efficiency, and minimal participant burden—making them especially suitable for use in aging populations or in large-scale community screenings.

However, this study has several key limitations that inform important directions for future research. First, the unequal distribution of participants across age groups—particularly the smaller sample size in the 60s cohort—may have limited statistical power and reduced the reliability of age-related comparisons, underscoring the need for more balanced sampling in future studies. Second, the relatively small overall sample size, especially within the cognitively impaired subgroup, constrains the strength of the conclusions and requires cautious interpretation of the findings as preliminary. Third, cognitive-status-specific analyses were restricted to the oldest cohort to minimize age-related confounding; therefore, future studies with larger samples should apply cognitive screening across all age groups to enable more comprehensive subgroup comparisons. Furthermore, Pearson correlation analysis was conducted across the entire sample despite the non-continuous age distribution, which may limit interpretation of age-related relationships; future studies should examine age-restricted correlations within older cohorts using larger and more evenly distributed age samples. Additionally, educational background was not systematically collected, which may limit the interpretive precision of MMSE-K-based cognitive subgrouping, although the primary physiological eye-tracking outcomes are less influenced by educational factors. Future studies should incorporate education-level data to strengthen cognitive classification. Moreover, statistical and cognitive-status-stratified pupillometry analyses were not performed, and future studies with larger, clinically diagnosed cohorts are needed to evaluate pupil dynamics in relation to cognitive impairment. Moreover, resting pupil diameter was not independently modeled in this study, although age-related reductions in baseline pupil size are recognized, and future studies should incorporate baseline pupil normalization to improve interpretation of pupillary dynamics. Although major confounding factors were screened and standardized testing conditions were applied, residual variability related to unmeasured physiological states (e.g., subtle fatigue or autonomic fluctuations) cannot be entirely excluded. Future studies should therefore incorporate larger, longitudinal cohorts with clinically diagnosed Alzheimer’s disease and related neurodegenerative conditions to determine whether combined microsaccade and pupil size variation can serve as sensitive and reliable markers of disease progression beyond age-related cognitive impairment.

## 5. Conclusions

This study explored the potential utility of simultaneously analyzing microsaccades and pupil size variations as non-invasive indicators of age-related cognitive changes. The findings are preliminary and exploratory, as the study employed a cross-sectional design and did not include clinically diagnosed Alzheimer’s disease patients; therefore, the proposed approach should be regarded as a potential screening framework rather than a validated diagnostic tool. Nevertheless, because aging and cognitive impairment are key risk factors along the Alzheimer’s disease continuum, the combined analysis of microsaccades and pupillary dynamics may offer a non-invasive, objective, and quantitative framework relevant to early Alzheimer’s disease research. By complementing traditional neuropsychological assessments such as the MMSE and MoCA, which are influenced by educational and task-related factors, these findings contribute foundational evidence for future longitudinal studies targeting neurodegenerative disorders.

## Figures and Tables

**Table 1:** Age-related pupil responsiveness.

Age	Constriction Rate (Left)	Constriction Rate (Right)	Dilation Rate (Left)	Dilation Rate (Right)
20s	−51.6%	−51.5%	114.9%	108.9%
60s	−43.7%	−44%	80.4%	81.1%
70s	−38.4%	−39%	63.4%	65.6%

**Table 2:** Age-related pupil light response speed.

Age	Constriction Time (s)	Dilation Time (s)
20s	1.22	7.6
60s	1.22	7.7
70s	1.13	7.5

## Data Availability

Data are contained within the article.
